# Biosynthesis and characterization of gold nanoparticles using Brazilian red propolis and evaluation of its antimicrobial and anticancer activities

**DOI:** 10.1038/s41598-021-81281-w

**Published:** 2021-01-21

**Authors:** C. E. A. Botteon, L. B. Silva, G. V. Ccana-Ccapatinta, T. S. Silva, S. R. Ambrosio, R. C. S. Veneziani, J. K. Bastos, P. D. Marcato

**Affiliations:** 1 GNanoBio, School of Pharmaceutical Sciences of Ribeirão Preto, University of São Paulo, Avenida Do Café S/nº, Ribeirão Preto, São Paulo, 14040-903 Brazil; 2 School of Pharmaceutical Sciences of Ribeirão Preto, University of São Paulo, São Paulo, Brazil; 3 Research Center of Exact and Technological Sciences, UNIFRAN, São Paulo, Brazil

**Keywords:** Cancer, Cell biology, Drug discovery, Microbiology, Plant sciences, Diseases, Chemistry, Materials science, Nanoscience and technology

## Abstract

Gold nanoparticles (AuNPs) are highlighted due to their low toxicity, compatibility with the human body, high surface area to volume ratio, and surfaces that can be easily modified with ligands. Biosynthesis of AuNPs using plant extract is considered a simple, low-cost, and eco-friendly approach. Brazilian Red Propolis (BRP), a product of bees, exhibits anti-inflammatory, anti-tumor, antioxidant, and antimicrobial activities. Here, we described the biosynthesis of AuNPs using BRP extract (AuNP_extract_) and its fractions (AuNP_hexane_, AuNP_dichloromethane_, AuNP_ethyl acetate_) and evaluated their structural properties and their potential against microorganisms and cancer cells. AuNPs showed a surface plasmon resonance (SPR) band at 535 nm. The sizes and morphologies were influenced by the BRP sample used in the reaction. FTIR and TGA revealed the involvement of bioactive compounds from BRP extract or its fractions in the synthesis and stabilization of AuNPs. AuNP_dichloromethane_ and AuNP_hexane_ exhibited antimicrobial activities against all strains tested, showing their efficacy as antimicrobial agents to treat infectious diseases. AuNPs showed dose-dependent cytotoxic activity both in T24 and PC-3 cells. AuNP_dichloromethane_ and AuNP_extract_ exhibited the highest in vitro cytotoxic effect. Also, the cytotoxicity of biogenic nanoparticles was induced by mechanisms associated with apoptosis. The results highlight a potential low-cost green method using Brazilian red propolis to synthesize AuNPs, which demonstrated significant biological properties.

## Introduction

Metallic nanoparticles can be considered one of the most versatile types of nanoparticles due to their applications in chemistry, electronics, medicine, and pharmaceutical sciences^1^. Among them, the gold nanoparticles (AuNPs) stand out for their advantages such as biocompatibility, tunable optical properties, and easily changed surface chemistry^2,3^. Because of these unique physical–chemical properties, the AuNPs are widely used as carriers of drugs and molecules to improve the diagnosis and treatment of diseases^4,5^.

The synthesis of AuNPs through chemical and physical routes has been already well-established. However, these pathways generally use toxic substances and non-polar solvents, which generate hazardous impacts for the environment and requires various steps of product purification, resulting in an expensive process^6^.

For overcoming the challenges related to conventional methods, a biosynthetic route has been proposed in the literature^7^. The green synthesis uses natural compounds from plants or microorganisms (e.g., fungi, bacteria, algae) as precursors of the reaction of gold ions reduction^8,9^. Biosynthesis is considered a simple, low-cost, and eco-friendly approach since it uses non-toxic solvents, such as water^10^. The production of metallic nanoparticles using natural sources has already been reported in the literature, showing it is a potential synthetic route that should be explored^11,12^.

Plant extracts are complex mixtures providing a rich arsenal of molecules with high redox potential^13^, such as flavanones, flavones, flavonols and chalcones, fatty acids, amino acids, terpenoids, aldehydes, and alcohols^14^. Furthermore, biogenic synthesis produces large amounts of highly stable nanoparticles with a better-defined size than some conventional methods since phytochemicals compounds that are used in the reaction also act as stabilizing agents^15,16^.

Propolis is a product of bees, known worldwide for its biological properties and used in traditional medicine. Propolis exhibits significant pharmacological activities such as anti-inflammatory^17,18^, anti-oxidant^19,20^ and antimicrobial^21,22^. It has also been demonstrated that propolis has anti-proliferative and anti-tumor effects in vitro and in vivo tumor models^23–25^.

There are several types of propolis with different compositions depending on the region, climate, and extraction season^26,27^. More than 300 substances have already been identified in different samples of propolis^28^. Some of the propolis types have been used to produce gold nanoparticles since this natural product presents a high amount of polyphenolic acids, flavonoids, terpenoids, and other molecules that can reduce Au^+3^ to Au^0^^29–31^.

Red propolis has been found in countries such as Mexico, Cuba, China and Brazil^32^. Brazilian red propolis (BRP) is found in northeastern Brazil^33^ and is considered to be a distinct type of propolis since it has some molecules of pharmacological interest that have not yet been discovered in other kinds of propolis^34–36^.

Several studies about the anticancer activity of red propolis have been described in the literature^37–39^. Researchers reported the cytotoxic and anti-proliferative effects of red propolis in cell lines of leukemia and prostate cancer^40^. Frozza et al.^41^ reported that red propolis promoted apoptotic effects in human cancer cell lines through mitochondrial perturbation.

Here, we investigated, for the first time, the use of Brazilian red propolis extract and its fractions in the biosynthesis of gold nanoparticles using an eco-friendly and low-cost method. This study also evaluated the structural properties of these nanomaterials and their biological activities against microorganisms and cancer cell lines.

## Results and discussion

### Preparation of Brazilian red propolis extract and its fractions

**Figure 1:**
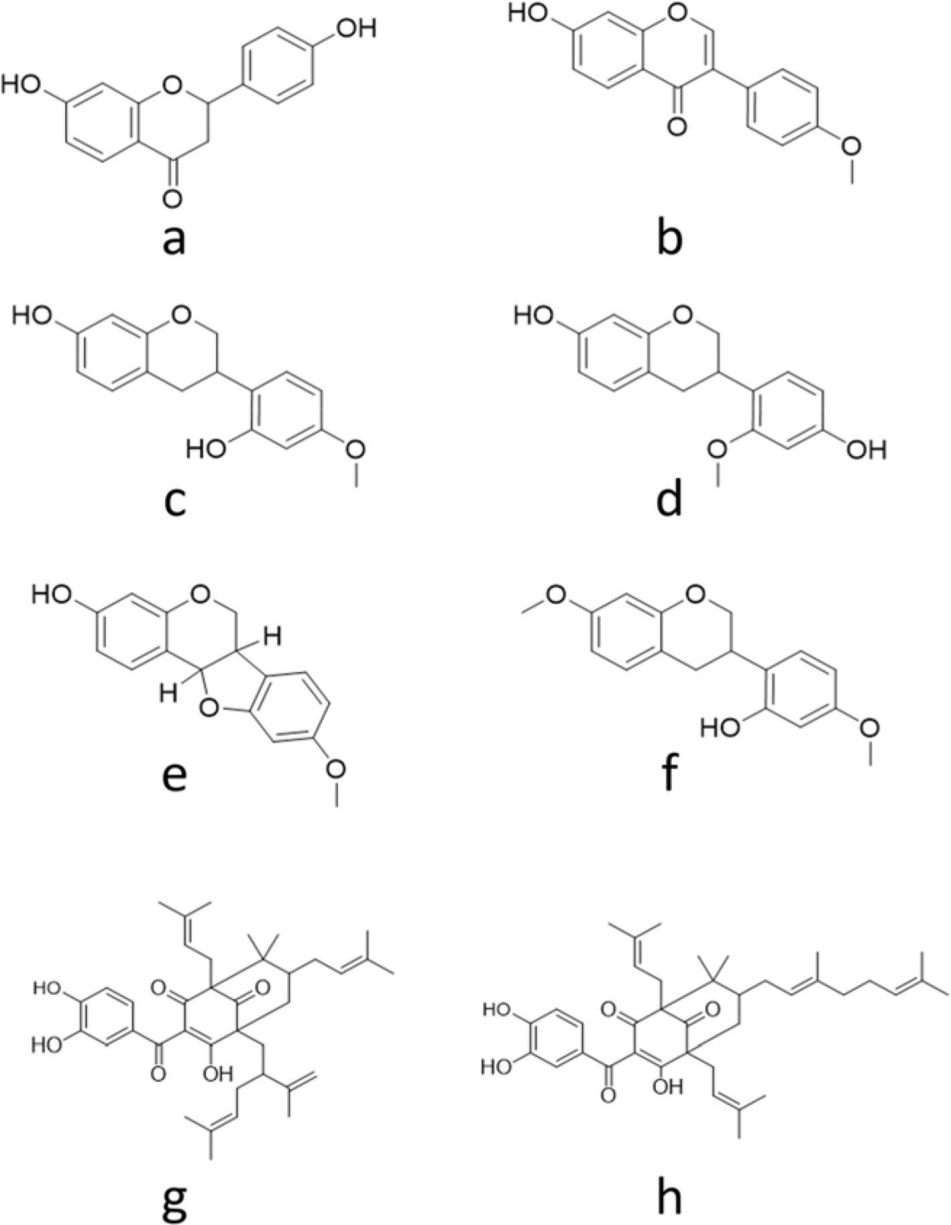
Chemical structures of the main constituents from Brazilian red propolis.

Brazilian Red Propolis (BRP) displays different biological activities such as antimicrobial, anticancer, antioxidant, and anti-inflammatory^34,35^. These activities are related to its complex chemical composition including liquiritigenin (a), formononetin (b), vestitol (c), neovestitol (d), medicarpin (e), 7-*O*-methylvestitol (f), guttiferone E (g) (Fig. 1). These biomolecules were identified in the chromatographic profile of BRP extract (Table 1). The solid-phase extraction of the red propolis hydroalcoholic extract left 23.8 g of the hexane fraction (27.3% yield), 54.2 g of the dichloromethane fraction (62.3% yield), 4.3 g of the ethyl acetate fraction (5%), and 3.6 g of the ethanol fraction (4.1% yield). The phytocompounds identified by HPLC in each fraction are shown in Table 1.

**Table 1:** Major phytochemical compounds identified in the BRP extract and its fractions by HPLC.

Compounds	Retention time	BRP extract	Hexane fractions	Dichloromethane fraction	Ethyl acetate fraction
Liquiritigenin	9.89	+	−	+	+
Formononetin	12.90	+	−	+	+
Vestitol	13.17	+	−	+	+
Neovestitol	13.72	+	−	+	+
Medicarpin	14.82	+	−	+	−
7-*O*-methylvestitol	17.22	+	−	+	−
Guttiferone E	25.69	+	+	+	−
Oblongifolin B	25.96	+	+	+	−

### Biosynthesis of gold nanoparticles

Au(III) has a high reduction potential and can be reduced by phenolic compounds from the natural extracts^42^. In this study, we produced gold nanoparticles (AuNPs) with BRP extract (AuNP_extract_) and its fractions (AuNP_hexane,_ AuNP_dichloromethane_, AuNP_ethyl acetate_) using the green synthesis method. In order to increase the solubility of the compounds present in the extract, the pH of the mixture of gold solution with the propolis extract was adjusted to 7.0. Moreover, this pH alteration is also involved in the activation of phytochemical compounds, which facilitates the donation of electrons to the metal, reducing Au^3+^ to Au^0^^42^.

**Figure 2:**
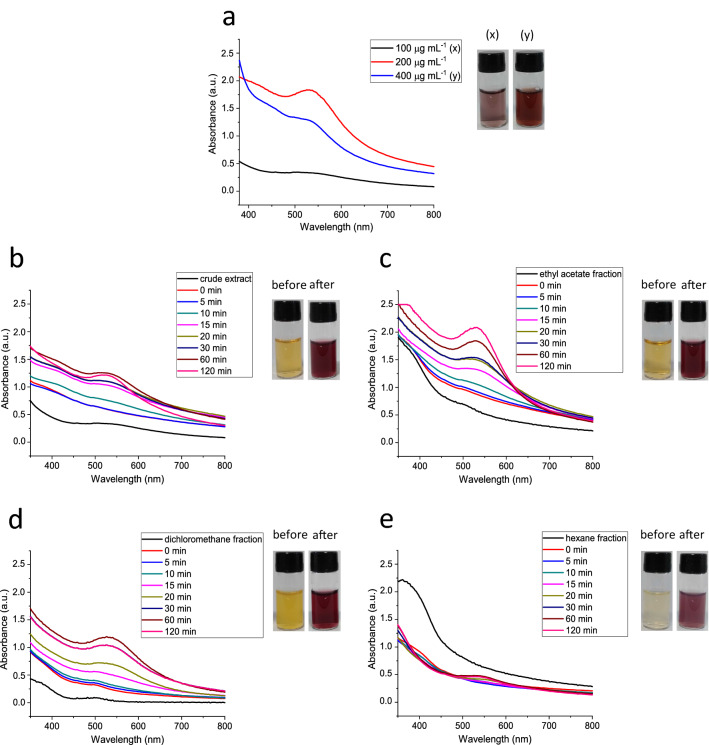
UV–vis spectra of biosynthesized gold nanoparticles: (**a**) using different BRP extract concentrations; (**b**) AuNP_extract_; (**c**) AuNP_ethyl acetate;_ (**d**) AuNP_dichloromethane_; (**e**) AuNP_hexane_.

The formation of AuNPs was confirmed by the presence of the Surface Plasmon Resonance (SPR) band. This band occurs on the surface of certain metals on a nanometer scale^43^. Both the extract and its fractions (hexane, dichloromethane and ethyl acetate) produced AuNPs, showing a prominent peak at a range of 523–541 nm (Fig. 2). The optimal extract or fractions concentration to produce AuNPs was also investigated. The high formation of AuNPs was obtained using 200 μg mL^−1^ of the extract or its fractions. Moreover, we verified a wider SPR band and turbidity signs in the AuNPs dispersion when the red propolis extract concentration was increased twofold (400 μg mL^−1^) (Fig. 2a).

In consonance with Gatea et al.^30^ and Roy et al.^29^, we also observed an increase in the absorbance values related to the time with a redshift of the SPR band (Fig. 2b–e). All formulations exhibited a similar absorption peaks profile, indicating a rapid growth of particles. After some time (~ 2 h), the saturation was reached, indicating the formation of stable nanoparticles^29^. The acetate and dichloromethane fractions were more efficient to produce AuNPs since the intensity of SPR bands was higher than the others^44^. This result can be associated with the higher polarities of dichloromethane and ethyl acetate in comparison with hexane used in the extraction process^45^. The color of the samples changed from pale yellow to dark red or purple (Fig. 2). The different colors of AuNPs—from light pink to dark red—are dependent on the size, shape, and structural characteristics of these nanoparticles^42^.

### Morphology and diameter distribution

**Figure 3:**
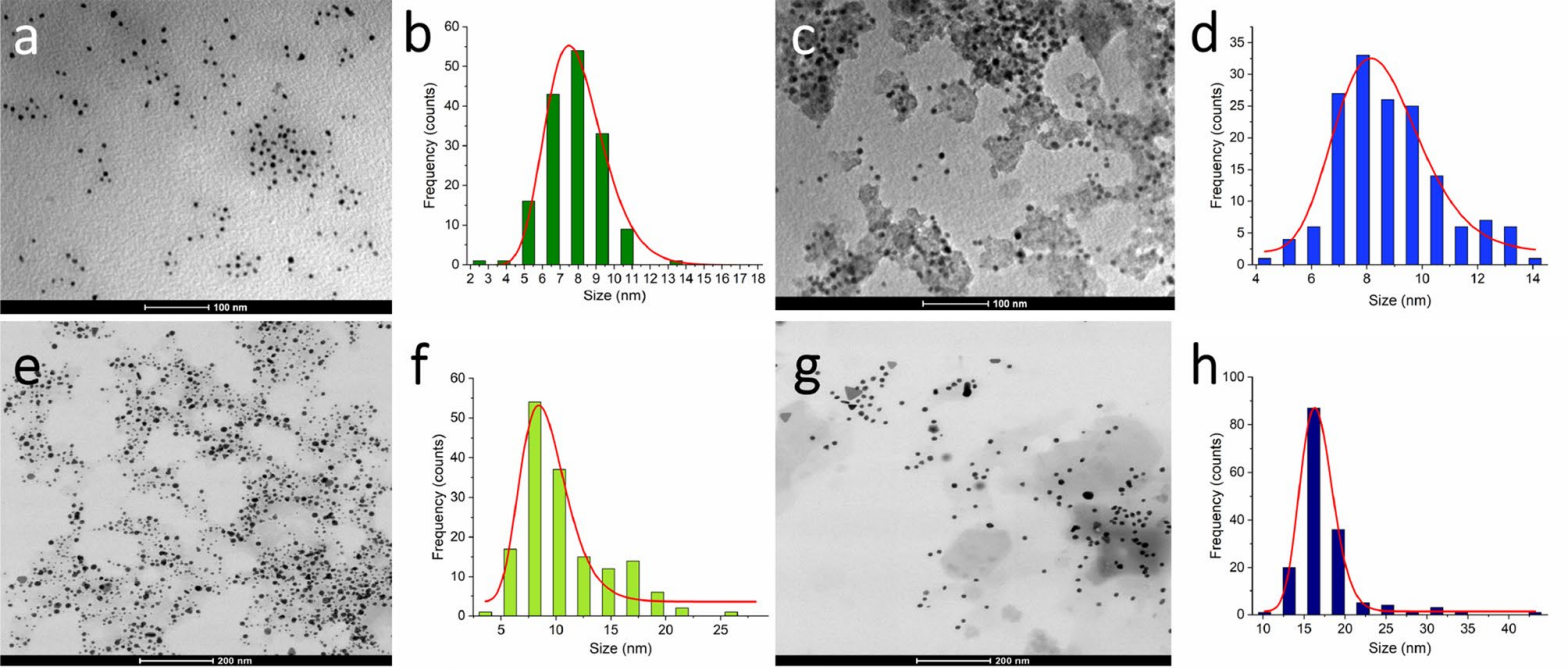
TEM images and size distribution histograms of biosynthesized gold nanoparticles: (**a**,**b**) AuNP_extract_; (**c**,**d**) AuNP_ethyl acetate_; (**e**,**f**) AuNP_dichloromethane_; (**g**,**h**) AuNP_hexane._

The morphologies and diameter distributions of AuNPs formed by three fractions and crude extract of BRP were investigated using the transmission electron microscopy (TEM) analysis. Figure 3a,c exhibits AuNP_extract_ and AuNP_ethyl acetate_ with mostly spherical shapes, whereas the AuNP_dichloromethane_ and AuNP_hexane_ showed a variety of shapes (Fig. 3e,g). In general, the biosynthetic route produces nanoparticles with different morphology and size as a result of the chemical composition of the extract^46,47^. Hexane fraction is rich in benzophenones (Guttiferone E; Oblongifolin A), while ethyl acetate fraction is rich in flavonoids and isoflavonoids such as liquiritigenin, formononetin, vestitol, and neovestitol. BRP crude extract and dichloromethane fraction present a similar phytochemical profile (Table 1).

**Figure 4:**
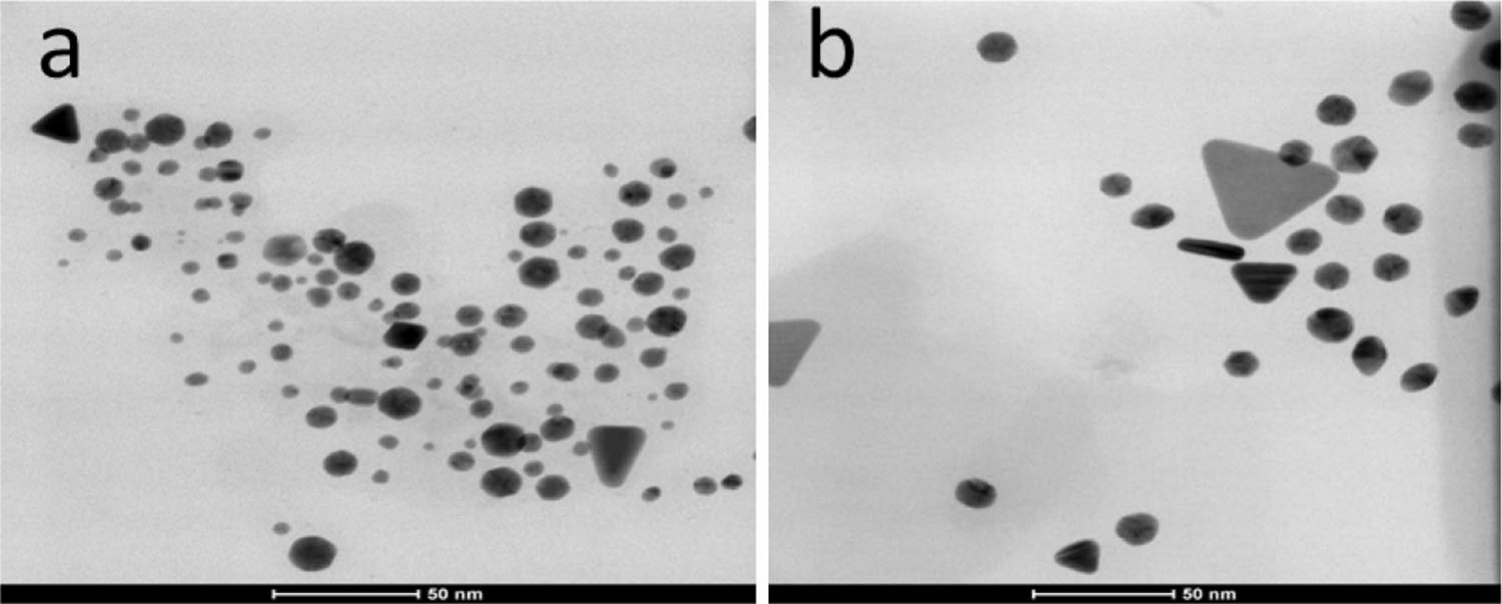
TEM images of (**a**) AuNP_dichloromethane_ and (**b**) AuNP_hexane_ showing the different shapes.

The average size, measured by TEM, was in the range of 8–15 nm for all gold nanoparticles showing a narrow particle size distribution (Fig. 3b,d,f,h). The smaller nanoparticles showed spherical shapes, whereas the larger particles exhibited several geometrical forms, such as triangles, pentagons, hexagons, and rods (Fig. 4a,b). Similar results were reported by Smitha et al.^48^, Philip et al.^49^, and Gosh et al.^50^, who verified that nanoparticles size with different geometries were larger than those with spherical morphology due to low quantities of the efficient biomolecules responsible for capping and stabilization, leading to the formation of large anisotropic nanoparticles^49^.

**Figure 5:**
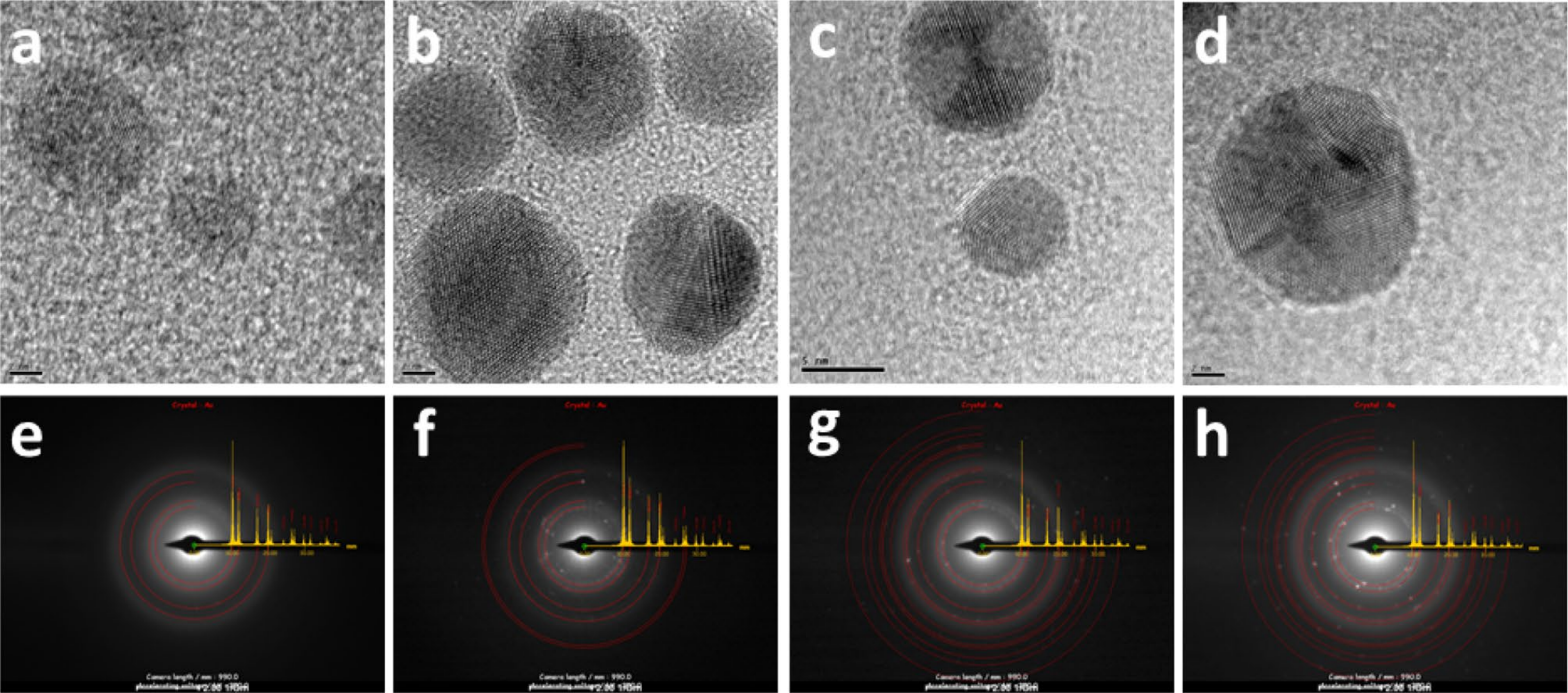
HR-TEM images and SAED pattern of biosynthesized gold nanoparticles: (**a**,**e**) AuNP_extract_; (**b**,**f**) AuNP_ethyl acetate_; (**c**,**g**) AuNP_dichloromethane_; (**d**,**h**) AuNP_hexane._

The Energy Dispersive X-ray Spectroscopy (EDXS) was performed to gather information about the chemical composition of samples for elements with atomic numbers (Z) > 3. All AuNPs spectra showed an absorption band peak of approximately 2.2 keV, which is characteristic of gold absorption^5,51^. The HR-TEM images (Fig. 5a–d) of all AuNPs demonstrated highly ordered planar spacing consistent with the internal spacing of the gold plane. The AuNP_hexane_ showed inter-planer spacing of 1.9 Å, whereas the AuNP_ethyl acetate_ and AuNP_dichloromethane_ exhibited 2.2 Å of d-spacing, which is in agreement with the (200) and (111) lattice of face-centered cubic (fcc) gold (JCPDS card N^o^ 04-0784)^52,53^. A crystalline structure of the biosynthesized gold nanoparticles was also evidenced by the SAED pattern (Fig. 5e–h) with circular rings that can be assigned to (111), (200), (220), and (311) Bragg’s reflection planes^54^.

### Fourier-transform infrared spectroscopy (FTIR) and Thermal gravimetric analysis (TGA)

The relation of phytochemicals compounds involved in the biosynthesis and stabilization of AuNPs was evaluated by FTIR and TGA. Table 2 shows the main peaks in the FTIR spectra of BRP extract or its fraction, as well as the AuNPs produced.

**Table 2:** Relation of the main peaks found in FTIR spectra of the BRP extract and its fraction and biosynthesized AuNPs.

Peak	Bands (cm^−1^)		Possible functional groups	References
	Extract and fractions	AuNPs		
1	3436; 3438; 3445; 3436;	3430; 3426; 3426; 3445;	Free O–H	Ismail et al.^59^; Park et al.^61^; Elbagory et al.^62^; Gatea et al.^30^;
2	2922; 2924; 2940; 2921;	2918; 2932; 2921; 2921;	CH_2_ stretching vibrations	Benedec et al.^10^; Liu et al.^60^; Gatea et al.^30^;
3	1729; 1713; 1729; 1732;	1729; 1729; 1739; 1689;	Carbonyl stretching vibrations	Benedec et al., 2018^10^; Liu et al., 2019;
4	1624; 1623; 1624; 1637;	1605; 1615; 1614; 1622;	Carboxylate anion –COO^−^	Benedec et al., 2018; Alexeree et al., 2017^63^;
5	1510; 1510; 1510; 1520;	1510; 1510; 1501; 1462;	Free NH groups	Ismail et al.^59^; Leon et al.^57^
6	Around 1380;		CH_3_ stretching vibrations	Elbagory et al.^62^;
7	Between 1280 and 1155;		Aromatic C–O bond stretching	Leon et al.^57^; Elbagory et al.^62^;
8	Between 890 and 775		C–H bonds in the phenolic rings	Liu et al.^60^; Zhang et al.^64^

The peaks around 1700 cm^−1^ are distinctive in hexane, and the dichloromethane fractions spectra are associated with carbonyl groups (C=O). These signals can be related to the major compounds in these fractions such as prenylated benzophenones^55^ and phenolic compounds^56^. The peaks at 1600 cm^−1^ are usually correlated to the stretching vibration of carboxylate anion –COO^−^, probably due to the oxidation of polyphenols during Au^+3^ reduction^10,57^. These bands show large amounts of alcohol or phenol in plant extracts.

**Figure 6:**
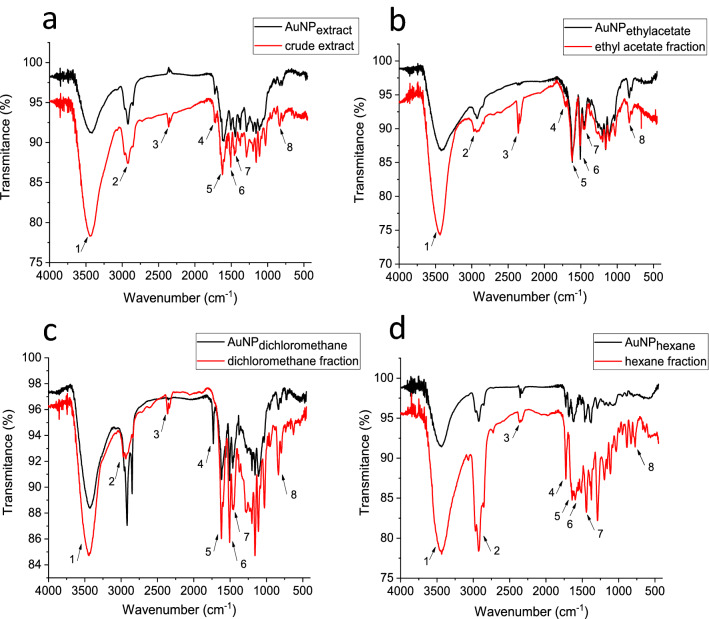
Fourier transform infrared (FTIR) spectra of the BRP extract (red line) and biosynthesized AuNPs (black line).

The bands around 3400 cm^−1^ in the AuNPs dispersions were smaller than the extract FTIR spectra (Fig. 6), suggesting the oxidation of hydroxyl groups to carbonyl groups. It indicates that the OH groups present in BRP extract or fractions are the main compounds involved in the reduction of Au ions^58^. The absorption peaks measured at 1500 cm^−1^ are probably due to free NH groups present in proteins. The reduction of this band intensity after the formation of AuNPs indicates that the proteins in the extract were also used for capping AuNPs, improving its stability^59^. Furthermore, decreased peak intensity at 800 cm^−1^ suggests the binding between the C-H group of phenolic acids and AuNPs^60^.

These results explain the role of chemical compounds of the BRP extract in reducing Au^+3^ and stabilizing AuNPs^65^. Also, FTIR spectra results support the idea that biosynthesized nanoparticles are surrounded by a thin layer of phytomolecules including polyphenols, such as flavonoids and tannins, in addition to terpenoids and proteins^58^. The BRP extract FTIR spectra show intense bands in 3436 cm^−1^ and 2922 cm^−1^, before and after the Au ions reduction (Fig. 6), which corresponds to free O–H bonds and CH_2_ stretching vibrations, respectively^30,59^.

**Figure 7:**
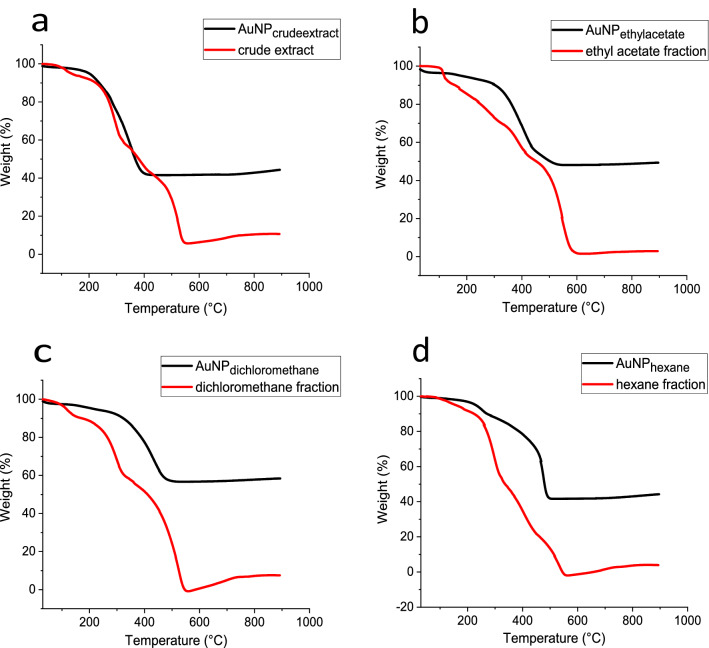
TGA analysis of BRP crude extract and its fractions as well as the biosynthesized AuNPs.

The TGA graphs of the BRP extract, fractions, and AuNPs (Fig. 7) showed a steady weight loss in the temperature range of 150–600 °C with a total weight loss of up to 800 °C. Table 3 shows the percentage of weight loss of the AuNPs. The percentage differences in the weight loss shown in the table are related to the difference in organic composition present on the AuNP's surface. It is expected that the weight loss between 100 °C and 200 °C refers to the evaporation of adsorbed water of the capping extract^8^. The largest weight loss observed in the range of 250 to 500 °C may be a result of the thin layer burning of organic material surrounding the nanoparticles^59^. Also, it is supposed that after 400 °C occurs the degradation of resistant aromatic compounds^62^. At the final of the thermal decomposition process, the residue of around 50% refers to the presence of pure AuNPs. These results indicate the protective effect of the extract compounds on the surface of AuNPs^66^.

**Table 3:** Weight loss percentage of the AuNPs.

Sample	Weight % up to 200 °C	Weight % up to 600 °C	Weight % up to 800 °C
AuNP_extract_	< 1.00	58.36	44.34
AuNP_ethyl acetate_	2.97	48.74	49.58
AuNP_dichloromethane_	2.39	40.87	58.41
AuNP_hexane_	10.68	47.57	44.24

### Antibacterial and antifungal activities

The MIC and MBC results are shown in Table 4. According to Aligiannis et al. (2001)^67^, plant materials with MIC up to 500 μg mL^−1^ are considered strong inhibitors of bacterial activity. Moderate inhibition is given by plant extract with MIC values between 600 and 1500 μg mL^−1^; whereas MIC above 1600 μg mL^−1^ is classified as weak inhibition. The BRP crude extract and the fractions hexane and dichloromethane showed a pronounced antimicrobial effect. No significant antibacterial activity of ethyl acetate fraction and AuNP_ethyl acetate_ was observed at the concentrations assessed. The AuNP_extract_ showed only fungicidal activity (MIC = 12.4 µg mL^−1^ of extract, equivalent to 0.2 × 10^9^ nanoparticles mL^−1^), whereas the AuNP_dichloromethane_ and AuNP_hexane_ demonstrated antibacterial and antifungal activity against all tested strains. AuNP_hexane_ exhibited the highest activity among the nanoparticles, showing MIC and MBC values similar to the values presented by the BRP crude extract (MIC = 50.8 and 101.7 µg mL^−1^ of extract equivalent to 0.5 and 1.9 × 10^9^ particles mL^−1^). Among the strains, *C. albicans* yeast showed the highest susceptibility, while *S. aureus* demonstrated the highest resistance to the AuNPs treatment.

**Table 4:** Minimum inhibitory concentration (MIC) and Minimum bactericidal concentration (MBC) of biosynthesized gold nanoparticles and BRP crude extract and its fractions against different microorganisms.

Results expressed in concentration of extract (µg mL^−1^)								
Samples	*Staphylococcus aureus*		*Escherichia coli*		*Streptococcus mutans*		*Candida albicans*	
	MIC	MBC	MIC	MBC	MIC	MBC	MIC	MBC
AuNP_extract_	> 198.6	> 198.6	> 198.6	> 198.6	> 198.6	> 198.6	12.4	12.4
AuNP_ethyl acetate_	> 234.5	> 234.5	> 234.5	> 234.5	> 234.5	> 234.5	> 234.5	> 234.5
AuNP_dichloromethane_	226.8	226.8	226.8	226.8	56.7	56.7	56.7	113.4
AuNP_hexane_	101.7	101.7	50.8	50.8	50.8	50.8	6.35	25.4
BRP crude extract	50.0	100.0	50.0	50.0	50.0	50.0	1.56	1.56
Ethyl acetate fraction	> 250.0	> 250.0	> 250.0	> 250.0	125.0	125.0	250.0	500.0
Dichloromethane fraction	62.5	125.0	125.0	> 250.0	15.64	31.25	31.25	62.5
Hexane fraction	15.64	31.25	62.5	62.5	7.82	7.82	3.91	15.64

BRP crude extract and its fractions exhibited the highest antimicrobial activity. In fact, the loss of activity of biosynthesized nanomaterials has been reported in the literature^30,68^. This difference might be explained by the oxidation of some active compounds of plant extracts during the reaction with metals. Several mechanisms have been proposed for the reduction of metal ions using plant extracts. Several authors suggest that the phenolic compounds are the main reducing and capping agents involved in the synthesis of metallic nanoparticles^69^. It would justify the lower antimicrobial activity of AuNPs, although they present phenolic compounds.

The difference between the antimicrobial activity of each biosynthesized AuNPs can be explained by the phytochemical composition of each extract and fraction^70^. According to a bio-guided study of Brazilian red propolis extract and its fractions, the crude extract and hexane fraction showed the highest antimicrobial activity against microorganisms, including *S. aureus* and *E. coli.* This activity can be related to the presence of medicarpin, elemicin, and vestitol^71,72^. Therefore, the medicarpin and vestitol we quantified in the BRP crude extract might have contributed to the antimicrobial effect of the BRP extract and AuNP_dichloromethane_.

Furthermore, benzophenones (Guttiferone E and Oblongifolin B) are present in low concentrations in the BRP crude extract and the dichloromethane fraction, and in high amounts in the hexane extract. Benzophenones are described as effective compounds against bacteria and fungi^39,73,74^. Also, the activity of AuNP_hexane_ and AuNP_dichloromethane_ can be related to the presence of these molecules on the surface of nanoparticles. On the other hand, AuNP_extract_ and AuNP_ethyl acetate_ showed low or no antimicrobial activity that can be associated with the low amounts of medicarpin, vestitol, and benzophenones in these extract and fraction. Moreover, ethyl acetate fraction did not present benzophenones showing the lower antimicrobial activity.

Additionally, the nanoparticle shape can influence its interaction with microorganisms and, consequently, affect its antimicrobial activity^75,76^. Thus, the heterogeneous gold nanoparticle shapes observed in the AuNP_dichloromethane_ and AuNP_hexane_ dispersion may have influenced its antimicrobial activity.

### Cytotoxic activity

The in vitro cytotoxic activity of BRP extract, fractions, and biosynthesized AuNPs were evaluated in bladder cancer cells (T24) and prostate cancer cells (PC-3) using the Resazurin assay. The resazurin assay provides a simple, non-toxic and sensitive measurement for the viability of mammalian cells^77,78^.

**Figure 8:**
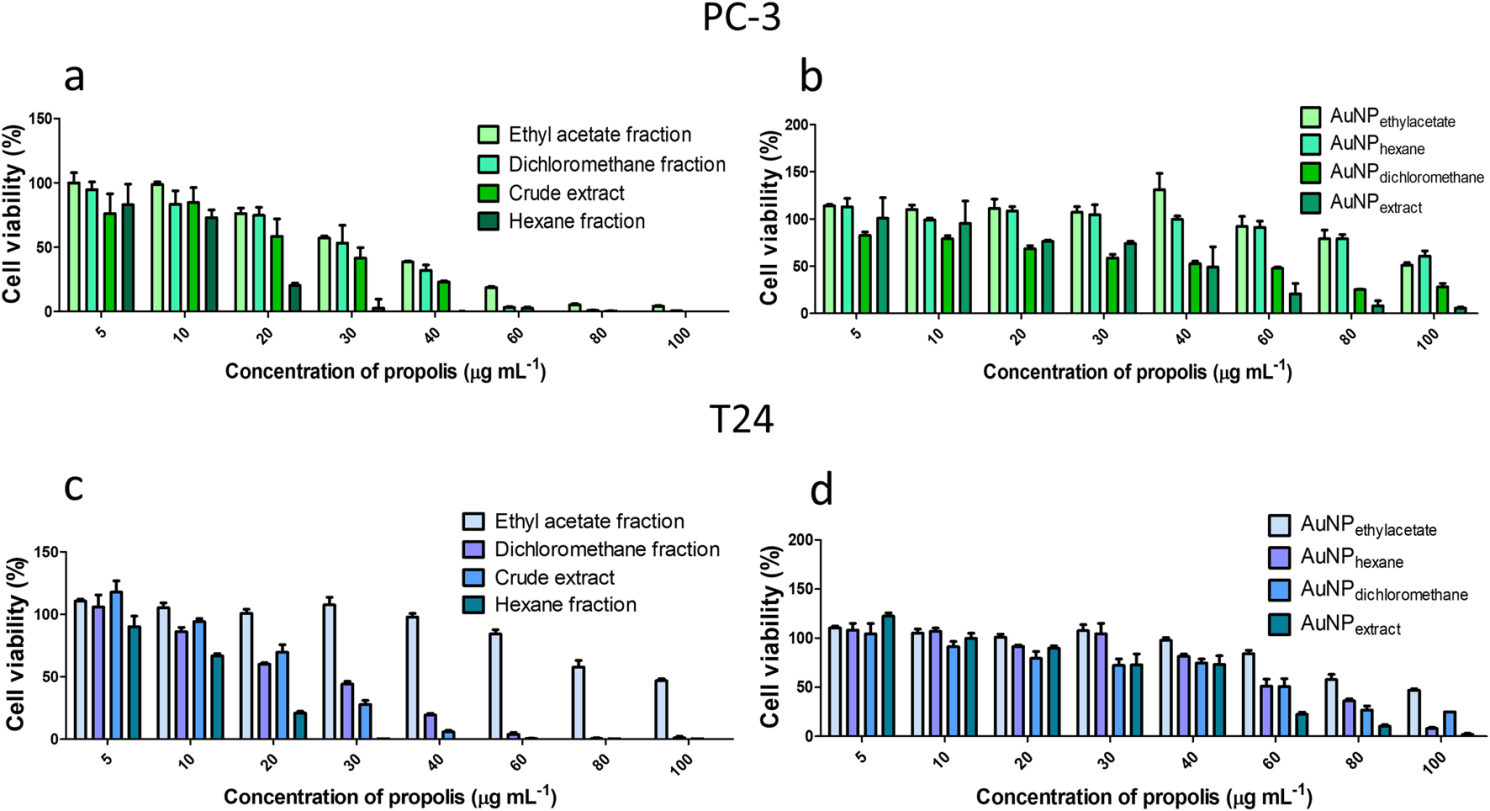
Cytotoxicity assay of extract and its fractions and biosynthesized gold nanoparticle in cells (**a**,**b**) PC-3 and (**c**,**d**) T24, obtained by resazurin assay.

Dose-dependent cytotoxic activity was observed in all samples (Fig. 8). The BRP crude extract and its fraction showed a high cytotoxic effect (Table 5). According to the U.S. National Cancer Institute, extracts with IC_50_ up to 30 µg mL^−1^ are classified as potent cytotoxic agents^79,80^. Moreover, BRP crude extract and dichloromethane fraction exhibited the highest cytotoxic effect (Table 5). BRP crude extract and dichloromethane fraction present similar phytochemical composition such as formononetin, liquiritigenin, and medicarpin in high concentration, which are known to exhibit cytotoxic activity in different cancer cells (Table 1)^81–83^. Ye et al.^84^ described that formononetin inhibited the proliferation of prostate cancer cells (LNCaP and PC-3) and induced apoptosis through the ERK1/2 MAPK-Bax pathway. Although AuNP_extract_ and AuNP_dichloromethane_ presented the same cytotoxic profile as their respective precursors (BRP crude extract and its fractions), the AuNP_extract_ exhibited the highest cytotoxic activity among the nanoparticles (Fig. 8 and Table 5).

**Table 5:** IC_50_ values of BRP crude extract, and its fractions and biosynthesized gold nanoparticles against T24 and PC-3 cancer cell lines.

Samples	IC_50_ (µg mL^−1^)	
	T24	PC-3
BRP crude extract	22.9	21.8
Ethyl acetate fraction	30.4	39.3
Dichloromethane fraction	22.1	26.1
Hexane fraction	11.6	12.5
AuNP_extract_	43.1	53.0
AuNP_ethyl acetate_	90.2	–
AuNP_dichloromethane_	48.5	63.0
AuNP_hexane_	59.5	89.0

The hexane fraction displayed the highest in vitro antitumor effects, with IC_50_ values around 12 µg mL^−1^ (Table 5), probably due to the presence of benzophenones (Table 1). Novak et al.^85^ reported that a red propolis fraction induced tumor regression of melanoma xenografts in mice and related this to a benzophenone’s activity. On the other hand, the low cytotoxicity of AuNP_hexane_ (IC_50_ of 59.5 µg mL^−1^ for T24 and 89 µg mL^−1^ for PC-3) might be explained by the participation of the benzophenones in the AuNP formation.

Ethyl acetate fraction and AuNP_ethyl acetate_ showed the least activity among the samples tested. These results could be related to the absence of compounds such as medicarpin and the benzophenones (present in extract and hexane fraction), which could have an important role in the anti-cancer activity and the synergism effect between the molecules. Some studies proposed that the anti-proliferative and cytotoxic effects of propolis against cancer cells might be correlated to the synergism between properties of several compounds and may not exclusively due to the concentration of a specific molecule^21^.

The difference in the IC_50_ values between PC-3 and T24 cells can be related to the malignant degree of the cells. T24 cell is derived from transitional cell carcinoma grade II, whereas the PC-3 cell line is derived from bone metastasis from grade IV adenocarcinoma. Thus, the PC-3 cell is more malignant and resistant to treatment^86^. Similar results were verified by Carvalho et al.^87^. Although all AuNPs were more cytotoxic for T24 cells, some nanoparticles showed high cytotoxicity to PC-3 cells decreasing the cell viability up to 10% in the highest concentration (Fig. 8).

Biogenic gold nanoparticles presented higher IC_50_ than their respectively extract or fraction precursors. These results can be explained by the mechanism of gold nanoparticles production, since some compounds of extract or fraction were used to reduce Au^3+^ to form the metallic nanoparticles in the green synthesis^29^. Hence, after nanoparticles preparation some active molecules of extract or fractions may lose its biological function. Owing to the complexity of propolis composition, future studies to determine the specific compounds from BRP that are responsible for gold nanoparticles reduction are demanded.

Although biosynthesized gold nanoparticles did not display a better cytotoxic effect than their precursors, exhibiting only residual cytotoxicity from BRP active compounds, they still constitute a great advantage compared with gold nanoparticles synthetized by chemical methods. El Domany et al.^88^ described that biosynthesized AuNPs were more cytotoxic than chemically synthesized AuNPs in studies with PC-3, HCT116, and HepG2 tumor cells. Besides, the easy modification of the allows their association with other antitumor drugs and molecules that can target specifics cells, such as cancer cells^89^. Researchers have been reported the beneficial effects of the combination between anticancer agents and polyphenols^90,91^, as well as compounds found in BRP^92–94^. Qiao et al.^95^ showed that green tea catechins associated with antitumor drugs are more effective than monotherapy.

### Adenosine triphosphate (ATP) bioluminescence assay

Cell culture viability was determined through quantification of the luminescent signal, produced by transformation of luciferin by luciferase as a function of intracellular ATP concentration^96^. ATP is the primary source of cellular energy, and its concentration is related to the number of living cells^97^. Luminescent ATP detection assay are robust and more sensitive than MTT or similar assays once the ATP generation depletes as soon as the cell dies^98,99^.

**Figure 9:**
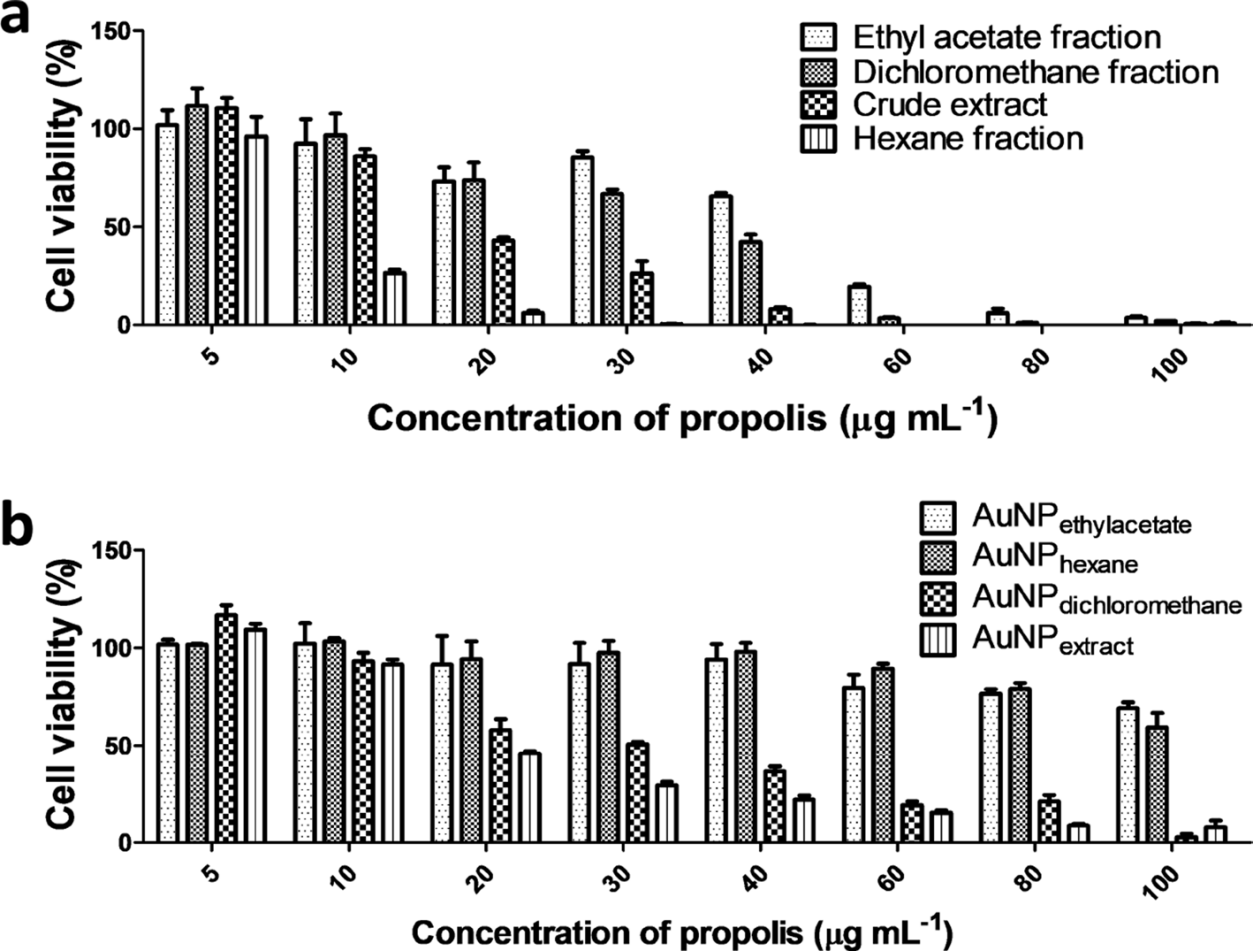
PC-3 cell viability of (**a**) biogenic nanoparticles (**b**) BRP crude extract and its fractions, obtained by ATP bioluminescence assay.

The results of ATP tests in PC-3 cells showed that biosynthesized AuNPs and its precursors (crude BRP extract and its fractions) present the same cytotoxic profile obtained by resazurin colorimetric determination (Fig. 9). This strain was chosen for the experiment because it is considered more resistant than T24 cells. Thus, as obtained in the resazurin assay, the hexane fraction was the most cytotoxic between the extract and fractions. Among the nanoparticles, AuNP_extract_ was the most effective in reducing cell viability, followed by AuNP_dichloromethane_. Therefore, these results confirm the cytotoxic profile previously verified by the resazurin assay.

### Flow cytometry

**Figure 10:**
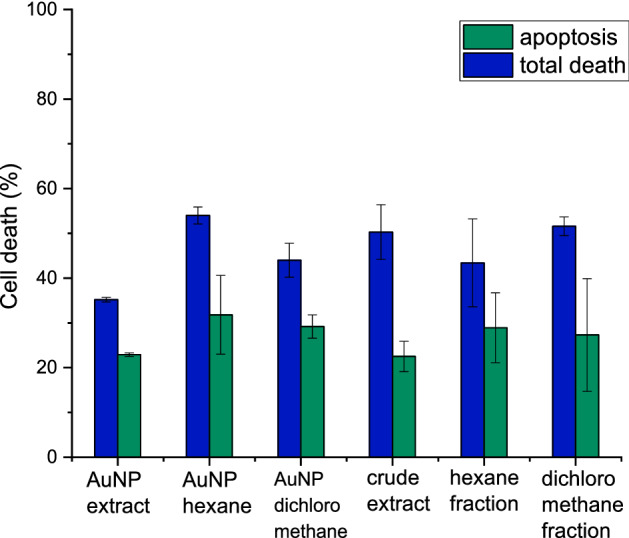
Percentage of apoptosis in relation of total PC-3 cell death obtained by Flow cytometry.

The cell death mechanism by flow cytometry assays demonstrated that BRP crude extract and its fractions, and biosynthesized AuNPs induced the death mainly by apoptosis. The percentage of apoptosis ranged between 44 and 66% related to the total of dead cells for all treatments (Fig. 10). Begnini et al.^100^ described BRP induced apoptosis in 5637 cells by molecular ways related to the P53 and Bax/Bcl-2. Another study conducted by Novak et al.^85^ demonstrated that BRP components induced cell cycle arrest of B16F10 in G2/M that triggers the apoptosis pathways activation.

This preliminary evidence has proven that biosynthesized gold nanoparticles using BRP displayed cytotoxic effects in cancer cells and can be a promising alternative therapeutic agent in cancer treatment.

## Conclusion

The Brazilian Red Propolis hydroethanolic extract and its fractions showed a great potential to produce gold nanoparticles with size range of 8–15 nm. Due to the specific composition of the extract or its fraction, AuNPs with different morphology were produced. Spherical AuNPs were obtained using BRP crude extract and ethyl acetate fraction, while dichloromethane and hexane fractions produced AuNPs with different shapes. All AuNPs showed a crystalline structure with a face-centered cubic (fcc) lattice. FTIR spectroscopy suggested the attachment of bioactive compounds from the Brazilian red propolis extract or its fraction on the surface of AuNPs. These results were confirmed by antimicrobial and cytotoxic activity of AuNPs produced. AuNP_extract_ showed antifungal activity and high cytotoxicity at low concentrations in bladder and prostate cancer cells. AuNPs obtained with dichloromethane and hexane fractions displayed high antibacterial and antifungal activities and cytotoxicity in both cells studied. On the other hand, the nanoparticles prepared with ethyl acetate fraction did not show antimicrobial activity and exhibited the lowest in vitro cytotoxic effect in the cells evaluated. The different phytochemical profiles of BRP extract and its fractions enabled the preparation of gold nanoparticles with distinct properties and interesting for several applications. Therefore, these in vitro results demonstrate the promising therapeutic applications of the biogenic gold nanoparticles in nanomedicine.

## Materials and methods

### Materials

Gold (III) chloride trihydrate (HAuCl_4_·3H_2_O), sodium hydroxide (NaOH), fetal bovine serum (FBS), penicillin/streptomycin, Resazurin sodium salt, dimethyl sulfoxide (DMSO), trypsin serine protease enzyme, Trypan Blue solution, tetracycline hydrochloride, and Amphotericin B solution were purchased from Sigma-Aldrich (Louis, MO, USA). The PC-3 prostate cancer cell line and the T24 bladder cancer cell lines were purchased from the Cell Bank of Rio de Janeiro (BCRJ). Dulbecco’s Modified Eagle Medium (DMEM), Roswell Park Memorial Institute medium (RPMI) and BD Brain Heart Infusion (BHI) agar were purchased from Thermo Fisher Scientific (Waltham, Massachusetts, USA).

### Collection of red propolis and preparation of crude extract

Red propolis sample collected in Canavieiras, State of Bahia, Brazil, was supplied by the beekeepers cooperative COAPER (Bahia, Brazil) in April of 2018. For the extraction, the propolis samples were frozen and grinded. Two hundred grams of red propolis were submitted to maceration with 70% hydroalcoholic ethanol solution in the ratio of 1:10 (w/v); propolis maceration was performed at 30 °C and 120 rpm using a shaker incubator (INNOVA 4300). The extracts obtained were concentrated under vacuum using a rotary evaporator and then lyophilized to complete dryness.

### Fractionation by the liquid–liquid partition of the crude extract of red propolis

The lyophilized red propolis crude extract (87 g) was subjected to a solid-phase extraction process. The extract was mixed with 200 g of microcrystalline cellulose, and the mixture was transferred to a 13 × 11 cm i.d glass column and submitted to successive extraction with organic solvents in increasing polarities: hexane (2 L), dichloromethane (2 L) and ethyl acetate (2 L). The fractions obtained were concentrated under vacuum and lyophilized to complete dryness.

### Biosynthesis of gold nanoparticles

AuNPs were synthesized by mixing a stock solution of tetrachloroauric acid trihydrate (0.5 mM) with BRP crude extract solution (200 μg/mL) or its fractions solution (hexane, acetate and dichloromethane), followed by NaOH addition until pH 7.0. The mixture was stirred for 1 h at optimized temperature. The gold nanoparticles formation was observed by color changed from pale yellow to dark red.

### Characterization of gold nanoparticles

The formation of gold nanoparticles was verified by the presence of a surface plasmon resonance (SPR) band, with maximum absorption between 500 and 550 nm, using UV–Vis spectroscopy. The spectra were collected at different times of formation using a UV–Vis spectrophotometer (Implen, Munich, Germany). AuNPs concentration used in the biological analysis was performed by Nanoparticle tracking analysis (NTA) using a Nanosight NS300 with 488 nm laser (Malvern Instruments). The morphology, shape, size and elemental composition of AuNPs were analyzed by Transmission Electron Microscopy (TEM) and high-resolution TEM FEI TECNAI G^2^ F20 coupled with Energy-dispersive X-ray Spectroscopy (EDXS) (Thermo Fisher Scientific, USA), operating a beam voltage of 200 keV. The diameter distribution was obtained using the Image J (NIH, USA) software using the TEM images. The crystalline structure of AuNPs was assessed by Selected Area Electron Diffraction (SAED). For Fourier-transform infrared spectroscopy (FTIR) and Thermal gravimetric analysis (TGA), biosynthesized AuNPs dispersion was centrifuged at 15,000 rpm for 60 min at 4 °C. AuNPs pellet was washed three times with deionized water with 50% ethanol to remove the excess of the extract from the AuNPs solution. The samples were lyophilized, and AuNPs powder or the BRP crude extract were mixed with potassium bromide (KBr) to obtain pellets. FTIR spectra were recorded using an IRTracer-100 (Shimadzu, Kyoto, JA) in the range of 4500 to 500 cm^−1^ with a resolution of 2 cm^−1^. TGA was carried out using an SDT Q 600 thermal analyzer (TA Instruments, New Castle, DE, USA). The samples were placed in platinum pans and heated under an inert atmosphere with a rate of 10 °C/min at 900 °C.

### Antibacterial and antifungal activities

Antibacterial and antifungal properties of biosynthesized nanoparticles were investigated using the Minimum Inhibitory Concentration (MIC) and Minimum Bactericidal Concentration (MBC) methods. The microorganisms used in this study belong to the American Type Culture Collection (ATCC) and are kept in the collection of the Applied Microbiology Research Laboratory (LaPeMA), University of Franca (UNIFRAN), under cryopreservation at  − 80 °C. Gold nanoparticles were tested against *Staphylococcus aureus* (ATCC 29213), *Escherichia coli* (ATCC 25922), *Streptococcus mutans* (ATCC 25175), and *Candida albicans* (ATCC 28366). MIC was determined by the broth microdilution method, in triplicate, at the exponential phase of bacterial growth using 96-wells microplates (CLSI 2007)^101^. The AuNPs samples were diluted in Brain Infusion Broth (Difco) and different concentrations of the samples (7.3 × 10^6^ to 7.5 × 10^9^ AuNP/mL) were added to each well. Then, the microorganism (5 × 10^5^ CFU/mL) was added to all wells. Tetracycline (0.0115 µg mL^−1^ to 5.9 µg mL^−1^) was used as a positive control for bacteria and Amphotericin B (0.031 µg mL^−1^ to 16 µg mL^−1^) was used as a positive control for yeast. The plates were incubated for 24 h at 37 °C. Afterward, a resazurin solution (0.02%) was added to determine the microorganism viability. Before Resazurin addition, an aliquot of the inoculum was removed from each well and seeded on BHI agar supplemented with 5% sheep blood for the MBC test. The plates were incubated as previously described. MBC was defined as the lowest concentration of the sample without microbial growth.

### Cytotoxicity assay

The cytotoxic activity of biosynthetic gold nanoparticles was evaluated in human urologic cancer cell lines. T24 bladder cancer and PC-3 prostate cancer cell lines were obtained from the Rio de Janeiro Cell Bank (BCRJ). T24 cell was cultured in RPMI medium (Sigma-Aldrich, USA) and PC-3 cell was cultured in DMEM-Dulbecco's Modified Eagle Medium (Sigma-Aldrich, USA), both containing 1% antibiotic (Penicillin–Streptomycin-Sigma-Aldrich, USA) supplemented with 10% fetal bovine serum (SBF-GIBCO, Thermo Fisher Scientific, USA). These cells were maintained in continuous culture under a humid atmosphere at 37 °C and 5% CO_2_. T24 or PC-3 cells were seeded in 96-well plates (2 × 10^4^ cells/well) and treated with different concentrations of biosynthesized AuNPs for 24 h. Afterward, the medium containing nanoparticles was removed and the cells were washed with PBS. Subsequently, a Resazurin solution (25 µg mL^−1^) was added to each well and incubated for an additional 4 h. Then, fluorescence was evaluated on a Microplate reader (Synergy HTX Multi-Mode Microplate Reader, Biotek) using 530 nm and 590 nm as the excitation and emission wavelengths respectively. The negative control received only medium and the positive control was treated with DMSO 1%. IC_50_ values were determined using the Graph Pad Prism version 8 software (GraphPad Software Inc. San Diego CA, USA).

### Adenosine triphosphate (ATP) bioluminescence assay

Cell viability was assessed using the CellTiter‑Glo Assay kit (Promega Corporation). The kit reagents were prepared according to the manufacturer's protocol. PC-3 cells were plated onto a 96-well plate at a density of 20,000 cells per well. The CellTiter‑Glo reagent was added to the washed cells 24 h after the treatments. The plates were shaken for 10 min at room temperature, followed by luminescence measurement using absorbance/fluorescence plate reader (Synergy HTX Multi-Mode Microplate Reader, BioTek).

### Flow cytometry

The percentage of apoptosis was determined by flow cytometry using FITC Annexin V (emission) as the apoptosis marker and FVD eFluor 450 (emission) as the viability marker. PC-3 cells were seeded in 6-well plates (6 × 10^5^ cell/well) and exposed to a concentration (IC_50_ values) of BRP crude extract and fractions and biosynthesized AuNPs. After 24 h, the cells were washed twice with phosphate-buffered saline (PBS) and incubated on ice with the FVD eFluor 450 for 30 min under dark condition. Then, cells were washed and marked with FITC Annexin V. After incubation with apoptosis marker, samples were acquired using a BD Accuri flow cytometer (Becton Dickinson). Gating of the viable cells and apoptotic cells was performed using the BD Accuri software.

### Statistical analysis

Statistical analysis was performed using the Prism 8 (GraphPad Software Inc. San Diego CA, USA) and Origin 2019 version (OriginLab, Northampton, Massachusetts, USA) software with significance level of 5% (p < 0.05). Results were analyzed by ANOVA followed by the Tukey post-test and data were expressed as mean ± standard deviation (SD).
